# CrossFit^®^: ‘Unknowable’ or Predictable?—A Systematic Review on Predictors of CrossFit^®^ Performance

**DOI:** 10.3390/sports11060112

**Published:** 2023-05-30

**Authors:** Nicole Meier, Jennifer Schlie, Annette Schmidt

**Affiliations:** Institut für Sportwissenschaft, Fakultät für Humanwissenschaften, Universität der Bundeswehr München, Werner-Heisenberg-Weg 39, 85577 Neubiberg, Germany

**Keywords:** CrossFit^®^, high-intensity functional training, functional fitness training, performance prediction, performance enhancement, physiological parameters, tactical athletes

## Abstract

The functional fitness training program CrossFit^®^ is experiencing fast-growing and widespread popularity with day-to-day varying ‘Workouts of the Day’ (WOD). Even among tactical athletes, the training program is widely applied. Nevertheless, there is a lack of data on which parameters influence CrossFit^®^ performance. For this reason, the purpose of this study is to conduct a systematic review of the existing literature to identify and summarize predictors of CrossFit^®^ performance and performance enhancement. In accordance with the PRISMA guidelines, a systematic search of the following databases was conducted in April 2022: PubMed, SPORTDiscus, Scopus, and Web of Science. Using the keyword ‘CrossFit’, 1264 entries are found, and 21 articles are included based on the eligibility criteria. In summary, the studies show conflicting results, and no specific key parameter was found that predicts CrossFit^®^ performance regardless of the type of WOD. In detail, the findings indicate that physiological parameters (in particular, body composition) and high-level competitive experience have a more consistent influence than specific performance variables. Nevertheless, in one-third of the studies, high total body strength (i.e., CrossFit^®^ Total performance) and trunk strength (i.e., back squat performance) correlate with higher workout scores. For the first time, this review presents a summary of performance determinants in CrossFit^®^. From this, a guiding principle for training strategies may be derived, suggesting that a focus on body composition, body strength, and competition experience may be recommended for CrossFit^®^ performance prediction and performance enhancement.

## 1. Introduction

The functional fitness training (FFT) program CrossFit^®^ (CrossFit, Inc., Washington, DC, USA) has rapidly developed into one of the fastest-growing training concepts, with over 15,000 affiliated training centers and 5 million athletes [1]. In the process, FFT continues to rank in the top 20 fitness trends of 2022 around the world [2]. Moreover, case reports are piling up about how the concept of CrossFit^®^ improves health or changes lifestyles [3,4,5].

As a result, there is increased interest in examining this fitness trend in more detail from a scientific point of view. To date, authors have examined a range of research areas surrounding the sport of CrossFit^®^ in several literature reviews [6]. The focus is usually on short- and long-term physiological responses [7,8,9,10,11], nutritional strategies or interventions [12,13], psychological parameters [14,15,16,17,18], and musculoskeletal injury risks of the training program [19,20,21,22,23,24]. Additionally, research on related training routines, such as high-intensity functional training (HIFT) [25] or high-intensity multimodal training (HIMT) [26] helps to determine how the training concept of CrossFit^®^ is practiced and applied. Taken together, CrossFit^®^ consists of a high-intensity training program, involving a high sense of community and motivational support with the overreaching aim to prepare athletes for any physical contingency, i.e., for ‘the unknown and the unknowable’ [27].

Thus, the training routine in CrossFit^®^ also reflects the demands for tactical populations to complete their missions. Tactical athletes include first responders (firefighters, police officers, paramedics, etc.) and military members, tasked with protecting the public. These occupations share that a reasonable level of physical fitness is required to ensure their readiness for unforeseeable challenges. Consequently, the training program of CrossFit^®^ is applied by several military and law enforcement units [25,28], so the impact of the application needs to be considered for this population as well.

In CrossFit^®^, the workouts involve constantly varied functional movements executed at high intensity. The day-to-day varying training routines are usually referred to as ‘Workout of the Day’ (WOD), and include exercises from the main elements of gymnastics (e.g., pull-ups, push-ups, and burpees), weightlifting (e.g., powerlifting, and Olympic weightlifting), and cardiovascular activities (e.g., running, rowing, and jumping) [29]. Workouts are typically scheduled to perform the required task as soon as feasible, i.e., ‘for time’ (FT), or to perform the maximum number of repetitions or rounds in a given time interval, i.e., ‘as many rounds as possible’ (AMRAP) [27]. Given the constant variation, it is critical to track performance changes through periodic monitoring of the performance of specific exercises. Therefore, Benchmark WODs are established to assess the progress of particular workouts, by comparing performance values at irregular intervals (e.g., number of repetitions and time to completion) over time or with other athletes [30]. The Benchmark WODs are standardized and must be performed under the same conditions to compare performance with athletes around the world, regardless of the location or environment in which they are performed. These workouts have names such as ‘Cindy’, ‘Fran’ or ‘Murph’ and a content of short and intense workouts (referred to as ‘Girl-WODs’), or long and hard to complete workouts (referred to as ‘Hero-WODs’) [31]. Physiological demands of the workout ‘Cindy’ (consisting of 20-min AMRAP of 5 pull-ups, 10 push-ups, and 15 air squats) [32,33,34], ‘Fran’ (consisting of FT of 21-15-9 repetitions of thrusters and pull-ups) [33,35], ‘Fight gone Bad’ (consisting of three rounds 1-min wall balls, 1-min sumo deadlift high-pulls, 1-min box jumps, 1-min push press, 1-min row, 1-min rest) [35,36], and ‘Murph’ (consisting of FR of 1-mile run, 100 pull-ups, 200 push-ups, 300 air squats, and 1-mile run) [37] are described in detail and have been studied so far. The programming of the most studied and well-known Benchmark WODs is additionally shown in Supplementary Table S1.

However, the focus of CrossFit^®^, independently of the standardized WODs, is on the variation of the training stimuli. In this way, athletes should achieve comprehensive fitness in order to optimally cope with any conceivable challenge, including unknown physical demands. In accordance, the constant variation of the training program is also evident in CrossFit^®^ competitions. Thus, CrossFit^®^ competitions differ from other sports by the characteristic that athletes are not supposed to know what to expect [38]. Otherwise, in sports events, the athletes know exactly which disciplines will be performed in the next competition. In contrast to the CrossFit^®^’s international competition, the ‘CrossFit^®^ Games’, where the WODs are not published in advance but are announced shortly before or even during the competition [39]. As a result, the athletes are unable to prepare specifically for a particular performance. The short-term announcement of the competition tasks thus represents a special feature of the ‘CrossFit^®^ Games’, causing them to be considered the ultimate fitness test worldwide. Since 2007, the ‘CrossFit^®^ Games’ have been held and annually awards the winners as ‘The Fittest on Earth^®^’ [39]. However, the athletes must first qualify for the ‘CrossFit^®^ Games’ in order to participate at the event. Currently, the ‘CrossFit^®^ Open’ represents the first level of the qualifying processes, feeding subsequent rounds in the competition system. From 2011 to 2018 the Regionals followed the ‘CrossFit^®^ Open’ as the second qualification phase. Due to several modifications to the competition’s structure in recent years, the regionals were replaced by quarter- and semi-finals in 2021 [40]. Participation in the ‘CrossFit^®^ Open’, meanwhile, is open to anyone at any performance level. In a recent study of the authors Mangine et al., normative scores for all ‘CrossFit^®^ Open’ workouts were collected and analyzed [41]. Accordingly, ‘CrossFit^®^ Open’ workouts are similarly used to compare and evaluate performance of CrossFit^®^ athletes. The ‘CrossFit^®^ Open’ workout descriptions of the years 2016 to 2020 are provided in Supplementary Table S1. Overall, the ‘CrossFit^®^ Open’ is considered one of the largest participatory sporting events, with more than 415,000 athletes signed up to compete in the year 2018 [42,43].

Despite the transformation into a worldwide public sporting event, to date, only a limited number of research approaches were carried out on the requirements for success in CrossFit^®^ competitions. In contrast to other individual or team sports, general performance-determining factors of CrossFit^®^-WODs are not yet known [44,45]. Since performance level in common sports (i.e., running, basketball, football, etc.) is usually quantified either by the athlete’s performance on the field or is implied by the athlete’s level of competition and years of experience [46], predictors can be inferred. On the other hand, the multifaceted and unknown demands of the workouts the changing nature of the past competitions, as well as the limited opportunity for athletes to gain specific competition experiences, complicate to identify success predictive factors in CrossFit^®^. Nevertheless, there are research approaches to determine appropriate predictor parameters that have a significant impact on CrossFit^®^ performance. Previous studies have investigated the influence of different factors on, for example, the performance of Benchmark WODs, of ‘CrossFit^®^ Open’ workouts or the placement in the ‘CrossFit^®^ Games’. By identifying these performance predictors, evidence-based recommendations for effective and specific training programming will be developed, leading to optimal competitive performance. However, there is still no overview and evaluation of the existing CrossFit^®^ predictor parameters. Therefore, the question remains whether a specific parameter exits that has a significant impact on CrossFit^®^ performance, regardless of the WOD performed. Furthermore, are the parameters applicable in the training routine for competition preparation or the preparation of tactical athlete missions? To answer these questions, a systematic review of performance predictors will contribute to.

In this regard, also tactical athletes can benefit from improved knowledge of predictive parameters of CrossFit^®^ performance. Since performance in CrossFit^®^ is quantified by coping with unfamiliar physical tasks, we hypothesized that predictors are also useful for the evaluation of the physical abilities of physical fitness of tactical populations. Considering that a number of sports tests are already used in professional environments, the review will also show whether they already incorporate predictors of CrossFit^®^ performance.

With limited evidence to date and no consensus on which parameters athletes should focus on most, CrossFit^®^ athletes, as well as tactical populations, still need to prepare for ‘the unknown and the unknowable’ [27]. To date, no systematic review has addressed this topic. Thus, the optimal preparation of athletes for the unpredictable demands of competitions remains a major challenge in science regarding FFT and the physical fitness of tactical athletes. In this context, this systematic review aimed to identify and summarize predictors of CrossFit^®^ performance and performance enhancement.

## 2. Methods

### 2.1. Study Design

To analyze the findings of scientific literature regarding predictors of CrossFit^®^ performance, a systematic literature search was conducted in accordance with the Preferred Reporting Items for Systematic Reviews and Meta-Analyses (PRISMA) guidelines [47,48]. In particular, this systematic review considers the question of what significant predictors of CrossFit^®^ performance and performance improvement are. Therefore, the research question adheres to the Population, Intertest and Context (PICo) strategy to determine relevant studies to include [49].

### 2.2. Study Eligibility

The inclusion and exclusion criteria are defined as the population includes healthy, adult participants of any gender (≥18 years), and studies on disease-state participants (e.g., overweight) are not considered. The focus of interest is on the determination of performance predictors in the context of competitive performance or performance enhancement of CrossFit^®^ athletes. Only research that reports predictive values based on its statistical data analysis is included in this review. Moreover, peer-reviewed research studies, and original research on humans written in English are eligible. The exclusion criteria are specific populations (children, seniors, people with disabilities), specific medical or nutrition interventions, non-CrossFit^®^-specific relations, duplicate articles, and not written in English. Additionally, articles that were systematic reviews, case reports or series, conference abstracts, dissertations, theses, and book chapters are not considered.

### 2.3. Search Strategy

**Figure 1:**
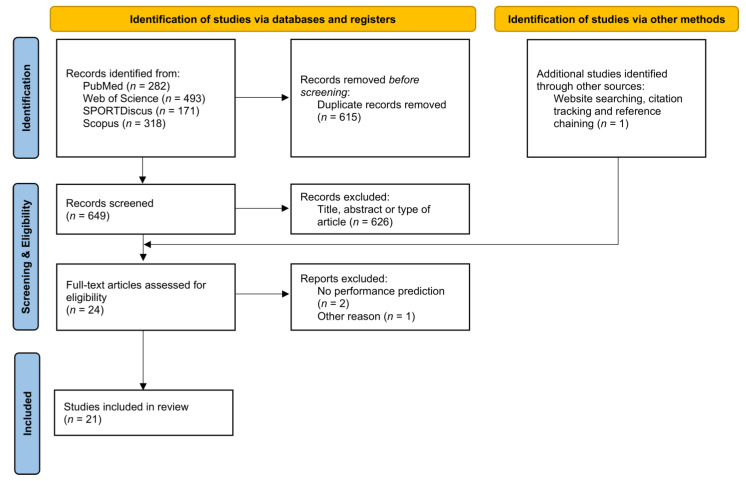
PRISMA flow diagram of the search strategy.

The systematic literature search was conducted in April 2022 using the following databases: PubMed, SPORTDiscus, Scopus, and Web of Science. Relevant articles were identified by using the search term ‘CrossFit’ without further restriction to obtain the maximum number of results. Search results were not limited to any particular number of years. To assure that relevant records are included, additional articles were identified through website searching, citation tracking, and reference chaining of relevant original and review articles, see Figure 1.

Subsequent to duplicate removal, two independent researchers (JS and NM) assessed the eligibility of the articles by reviewing the title and abstract of each record for inclusion and exclusion criteria. In a second phase, the articles were read in full text and selected for inclusion in this systematic review by the same two researchers (JS and NM) based on the eligibility criteria. If disagreements arose regarding the inclusion of articles, the expertise of a third reviewer (AS) was consulted to resolve the differences.

### 2.4. Data Items and Collection Process

Data extraction was performed by two researchers (JS and NM), followed by cross-checking and verification by a third researcher (AS) to avoid errors and reporting bias. Information on the author and year of publication, study design, participant′s characteristics, sample size, data collection, CrossFit^®^ performance, and main conclusions were extracted using standardized spreadsheets. In detail, significant predictor values for specific predicted performance with corresponding R-Squared or correlation coefficients were extracted, see Table 1.

The Tibana test (local muscle endurance test) consists of four following rounds with 2 min of rest between the rounds: 4 min of as many rounds as possible (AMRAP) of five thrusters, and 10 box jump over (round 1); 4 min of AMRAP of 10 power cleans, and 20 pull-ups (round 2); 4 min of AMRAP of 15 shoulder-to-overhead, and 30 toes to bar (round 3); and 4 min of AMRAP of 20-calorie row, and 40 wall balls (round 4).

Abbreviations: countermovement jump (CMJ); for time (FT); lean soft tissue mass index (LSTMI); repetitions (reps); relative strength index (RSI); squat jump (SJ); maximum oxygen consumption (VO_2max_); Wingate anaerobic test (WanT).

## 3. Results

### 3.1. Study Search

During the systematic search, 1264 titles were identified in the databases. First, 615 records that contained duplicates were excluded. Additionally, one article was included based on reference lists and article chaining and subjected to the full-text review. After reviewing the titles and abstracts of 649 articles, a total of 24 articles (0.04%) underwent full-text review to assess eligibility. Overall, 21 articles meet the inclusion criteria and are included in the systematic review. These articles are all published between 2015 and 2022 and written in English.

### 3.2. Performance Prediction and Enhancement

The majority of the includes studies focus on determining variables associated with performance or ranking at the ‘CrossFit^®^ Open’ or ‘CrossFit^®^ Games’ held in the years 2016 [50,64], 2017 [60], 2018 [51], 2019 [53,61,67], and 2020 [40,62]. Additionally, the common Benchmark performances (‘Cindy’, ‘Donkey Kong’, ‘Fran’, ‘Grace’, ‘Isabel’, ‘Kelly’, ‘Murph’, and ‘Nancy’) [37,55,56,58,59,61,63,65,66,68], as well as other usually performed WOD modalities (AMRAP or FT), are investigated [54,57]. The performance of the WOD ‘Fran’ is considered most frequently in seven studies [37,55,56,61,63,65,66]. Thereby, the sample size ranges from 10 to 32 participants when experimental measurements were used for data collection [37,50,51,53,54,55,56,57,58,59,60,61,62,63,65,66], and with the use of data available online [40,64] or reported data from questionnaires [52,67,68], the sample size went up to 220 [40]. However, upon further consideration, it is unfortunate to note that approximately half of the experimental data acquisitions only include male athletes in the investigation [37,40,53,54,56,59,60,63,65,66,67]. In this regard, Table 1 provides an overview of the parameters that significantly predict or are correlated with CrossFit^®^ performance. Overall, the studies aim to determine factors that are significantly correlated with CrossFit^®^ performance or that explain most of the variance by using Pearson’s and Spearman’s correlation, respectively, [37,40,62,63,64,66,67], and linear and multiple regression analysis [50,51,52,53,54,55,57,58,59,60,61], or principal component analysis [56,65].

In the study of Mangine et al., different pacing strategies for how to approach the challenges of the ‘2016 CrossFit^®^ Open’ explain the most variance. The results suggest that when the WODs consist of multiple rounds, competitors may employ a fast and sustainable pace to improve performance, and otherwise focusing on one or two key exercises is recommended [50]. Further, several studies on predicting CrossFit^®^ performance indicate that physiological parameters and high-level competitive experience influence more than one specific fitness marker [37,40,51,54]. In this context, the results of different authors providing no consensus on the relationship between the performance in Benchmark WODs (‘Cindy’, ‘CrossFit^®^ Total’, ‘Donkey Kong’, ‘Grace’, ‘Fran’, ‘Isabel’, ‘Kelly’, Murph’, and ‘Nancy’,) and selected exercise parameters [55,56,58,59,63,65]. The results show conflicting results which individual performance variable (e.g., 2000 m row, back squat, clean and jerk, pull-ups, sit-ups, snatch, or thrusters) is most important to achieve the best results in any WODs. Nevertheless, in three investigations, strong total body strength (i.e., CrossFit^®^ Total performance) indicates to be useful for higher workout scores [55,59,61]. In this manner, also the back squat performance explain variances of multiple parameters, such as the performance of the ‘CrossFit^®^ Open 2017′ [60], Fran [58,65], and snatch and clean and jerk [52].

Further, several authors analyze the influence of physiological parameters on the performance of common WODs or of the ‘CrossFit^®^ Open’ [37,51,53,57,58,61,65]. The correlations revealed the strongest association in increased performance with body composition [37,51,65], followed by aerobic capacity (maximum oxygen consumption (VO_2max_)) [53,61] and anaerobic power (Wingate anaerobic test (WanT) performance) [57,58]. So, the authors Mangine et al., demonstrate that body fat percentage (or body density) was the most important factor for success in the ‘2019 CrossFit^®^ Open’ [51]. However, the level of CrossFit^®^ experience also appears to be influencing, as indicated by the findings of the authors Bellar et al., that athletes’ experience is overall a consistent predictor of performance [54]. In particular, under competition conditions, the results of two studies by Mangine et al., also highlight that participation (i.e., experience in CrossFit^®^ competition) and ranking in previous ‘CrossFit^®^ Open’ were the most common predictors of the ‘CrossFit^®^ Open’ performance in the years 2018 and 2020 [40,51].

In relation, further approaches use specific testing methods to predict athletes’ performance. [57,62]. The authors Feito et al., note that the ability to quickly recover between high-intensity exercise units, as measured by the WanT, is positively related to performance in a 15-min AMRAP workout [57]. For the same reason, the authors Tibana et al., applied a specific local muscle endurance test (referred to as Tibana test) consisting of four following rounds with 2 min of rest between the rounds, see Table 1. The application demonstrated that the Tibana test and strength were strongly related to ‘CrossFit^®^ Open 2020′ performances [62]. However, in contrast to previous studies, body fat percentage [37,51] and cardiorespiratory capacity [53] are not significantly correlated [62]. Another factor influencing performance enhancement and mentioned in CrossFit^®^-related scientific literature concerns also the sleep quality of the athletes. A survey of 149 participants shows that CrossFit^®^ athletes with high sleep quality (determined by the Pittsburgh Sleep Quality Index) reported higher scores on all performance-related outcomes, particularly in the ‘Hero-’ and ‘Girl-WODs’ [68].

## 4. Discussion

### 4.1. Key Findings

This systematic review shows that no specific key parameter predicts CrossFit^®^ performance regardless of the type of workout and among the included studies, no consensus exists on the identified performance determinants. Overall, several different predictor values are found in the up-to-date literature to forecast CrossFit^®^ performance. However, the findings indicate a more consistent impact of physiological parameters, as body composition and aerobic capacity, as well as high-level competitive experience, in comparison to specific performance variables. One-third of the studies identified that high total body strength (i.e., CrossFit^®^ Total performance), and back squat performance correlated with higher workout scores.

This review includes 21 studies published between 2015 and 2022, highlighting the topicality of this research field. Despite the great discrepancies in the identified determinants of CrossFit^®^ performance, several variables are stated across different works and will therefore be discussed in detail. First of all, body composition (stated as body fat percentage and suprailiac skinfold thickness) was highly predictive of ‘CrossFit^®^ Open’, ‘Murph’, and ‘Donkey Kong’ performance in three studies [37,51,58]. CrossFit^®^ workouts include numerous bodyweight exercises on the power rack (e.g., pull-ups and muscle-ups) and the ground (e.g., push-ups and squats). Additionally, the ‘Murph’ challenge comprises two miles of running. This finding is therefore comprehensible and was earlier reported in related sports such as running [69]. Additionally, in corresponding literature on body weight workouts such as pull-ups, fat mass was identified as a determinant factor [70]. Next to that, aerobic capacity (VO_2max_) showed a moderate to high correlation with CrossFit^®^ performance in five of the studies [53,54,58,61,65]. Benefits of a high individual VO_2max_ were earlier reported in related aerobic sports such as triathlon [71,72]. As many CrossFit^®^ workouts include aerobic tasks such as running, cycling, swimming, or rowing, these findings are intelligible. Moreover, back squat performance was identified as a predictor across five different studies with moderate to high correlations [52,56,58,60,65]. In line with this, three studies reported a moderate to high correlation of strong total body strength (i.e., CrossFit^®^ Total performance) with performance in the ‘Grace’ and ‘Fran’ workouts [55,59,61]. Given that CrossFit^®^ combines elements from weightlifting, powerlifting, and other loaded tasks, this correlation is coherent. The back squat, deadlift, and chest or shoulder presses represent central elements of many CrossFit^®^ benchmark workouts. Therefore, great upper and lower body strength as well as good technique in these routines potentially represent crucial performance determinants. In particular, for WODs of Olympic weightlifting lifts (e.g., ‘Grace’), the impact of back squat performance is consistent with previous studies of the relationship of maximum strength to weightlifting performance [73]. Further, WanT performance was highly predictive of competitive CrossFit^®^ performance across three different studies [54,57,58]. The WanT tests the 30-s all-out sprint performance on a bike, and therefore resembles many CrossFit^®^ workouts that include short bike sprints for calories such as ‘The Climb’ or ‘Sneak Attack’.

Taken together, the above-mentioned determinants for competitive CrossFit^®^ performance are supported by research in resembling sports. The authors Ince and Ulupinar, for instance, investigated competitive performance prediction in young weightlifters and identified WanT performance, countermovement jump, and body fat percentage as best predictors [74]. Further, similar correlations between VO_2max_ and race performance can be found in endurance disciplines such as sprint triathlon [72]. Apart from physiological parameters and skills that have to be developed prior to a competition, one study identified the impact of different pacing strategies during the ‘2016 CrossFit^®^ Open’ on performance variance. For WODs consisting of multiple rounds, they recommend athletes to employ a fast and sustainable pace [50]. This pacing strategy is known from traditional continuous disciplines such as running and cycling, where consistent pacing was associated with faster race results [75]. Given the high diversity and varying intensity across many CrossFit^®^ workouts, identifying an efficient pacing strategy and sticking to it might however be harder compared to monotonic sports such as triathlon. Another work identified the correlation of previous CrossFit^®^ Open and Games ranks as well as regional appearances with recent competitive performance [40]. Corresponding literature supports this determinant in sports such as full-distance triathlon, where previous marathon race times predicted marathon times during an ironman event [76].

### 4.2. Recommendations and Strategies

Taking these findings into account, we can conclude a broad strategy for athletes seeking to improve their overall competitive CrossFit^®^ performance. In the weeks and months preceding a competition, simple factors such as reducing body fat percentage should be considered first. In particular when time constraints come into play, this could serve as an effective short-term intervention. Body weight workouts are easier to perform with less body fat, and therefore presumably lower total body mass. Moreover, lower fat mass directly influences relative VO_2max_ (ml/min/kg), thereby enhancing aerobic capacity without the need for aerobic exercise. As a second step, improving VO_2max_ itself through aerobic exercise could be a promising strategy. Several studies from different disciplines such as rowing, cycling, triathlon, and football showed, that already 1–8 weeks of high-intensity interval training can lead to significant improvements in aerobic capacity [77,78,79,80]. In particular in rather immediate competition preparation, the VO_2max_ should be addressed. Further, athletes should consider their one repetition maximum in weightlifting movements such as the back squat, deadlift, and shoulder press and compare their capabilities to high-ranked athletes of past competitions. This provides helpful orientations on whether improving this parameter could be of value for performance enhancement. On competition day, athletes should consider a proper pacing strategy for the posed challenges. In general, high sleep quality should be ensured, especially when competitions include technical and cognitive-demanding skills [68].

### 4.3. Application in Tactical Populations

Given the unpredictable tasks required to accomplish their missions, we hypothesized before, that CrossFit^®^ performance predictors could be transferrable to the evaluation of physical capabilities in tactical populations. Selection procedures and assessments of the physical performance of law enforcement, firefighting, and military personnel often incorporate physical fitness tests. The basic fitness test of the federal armed forces of Germany, for instance, consists of a pendulum sprint, a pull-up variation (sustained pull-up), and a 1000 m run [81]. Anthropometric measures such as body fat percentage or specific strength parameters are however not routinely evaluated outside of research settings. The U.S. Army combat fitness test includes six events. A three-repetition maximum of the deadlift, a standing power throw, a hand-release push-up, a sprint-drag-carry using a weighted sled and kettlebells, a plank hold, and a two-mile run [82]. Even though this test battery covers diverse physical challenges including specific strength parameters, anthropometric measures are still not considered. The results of this review however identified the correlation of body composition with CrossFit^®^ performance, which might be applied to the demands of military and law enforcement units. Therefore, the incorporation of these parameters could potentially complement a holistic selection procedure and physical fitness assessment in tactical populations.

### 4.4. Future Directions

A few research gaps are identified in this review. One factor that is barely addressed by recent CrossFit^®^ studies is the athletes’ sports history. The novelty of this exercise modality suggests that many of the top-performing athletes might have practiced other sports before engaging in CrossFit^®^. Which sports history might be especially promising for success in competitive CrossFit^®^ is a question that still needs further attention. Another gap that was identified is the impact of sponsoring or the economic set-up of an athlete. To our knowledge, no study to date tested, whether competitive success in CrossFit^®^ correlates with the available financial support or a given infrastructure and equipment. In particular, in times of increased home-based training due to the COVID-19 pandemic, this would be an interesting factor to consider [8]. In addition, to predict CrossFit^®^ competitive performance it would be valuable to address, how much a given athlete varies in performance from one event to another. In this context, a study by Malcata et al., analyzed within-subject variability for competitions within and between seasons. They identified a lower within-subject variability for endurance sports (0.6–1.4% variability between competitions, 1.0% for rowing) in comparison to sports that require explosiveness in a single skill execution, such as weightlifting (1.4–3.3%) (30). As CrossFit^®^ comprises both endurance as well as weightlifting elements, a comparable performance variability can potentially be expected. As underlying factors of performance variability, they addressed power output, environmental factors such as weather and audience, race dynamics and opponents, skill, and subjective scoring [83]. In CrossFit^®^, some of these factors apply. Regularly, either specific parts or a whole competition is carried out outside, so that wind and weather conditions matter. Further, changing audiences and opponents potentially impact competitive performance as well. Subjective scoring might however be limited in CrossFit^®^, as judgment follows clear specifications (FT or AMRAP) which leaves little room for subjectivity. Further, the brand makes sure to only employ certified CrossFit^®^ judges. When analyzing competitive performance in CrossFit^®^ these factors should therefore be considered. Thus, the within-athlete variability nevertheless raises an interesting research gap in this emerging field and should be investigated in more detail in the future.

### 4.5. Limitations

This analysis revealed several points that should be considered when applying the results into practice. At first, the poor comparability of the included studies was striking. Subjects of different skill levels were included, ranging from CrossFit^®^ novices to world-class athletes. Further, the workouts and competitions used for performance prediction varied. Some studies investigated the performance at the ‘CrossFit^®^ Open or Games’ for several days to weeks with a variety of workouts performed. Whereas many others analyzed single-benchmark workouts such as the ‘Fran’, which only lasts a few minutes and tests two specific exercises (thrusters and pull-ups). The latter studies can be misleading when it comes to overall performance at competitions such as the ‘CrossFit^®^ Games’. For this reason, single-workout studies might show limited explanatory power in comparison to a holistic analysis and raise the question of how well we can compare their results and draw conclusions. Apart from that it is legitime to ask, whether results from laboratory or field tests can at all directly be translated into performance in an official competition such as the ‘CrossFit^®^ Games’. Another limitation is the low number of females included in the studies. In the above-mentioned study by Malcata et al., the largest differences in within-subject variability by gender among all 16 different sport disciplines investigated were reported for weightlifting [83]. Female weightlifters exhibited a variation coefficient of 3.3% compared to 1.7% for males. Additionally, in sports such as triathlon, gender differences in performance determinants were earlier reported. Lower body fat percentage, for instance, was associated with enhanced race performance in male long-distance triathletes but not in females [84]. These examples underline the importance of including both genders in research on CrossFit^®^ performance, in order to make findings generalizable for all athletes.

## 5. Conclusions

For the first time, this review presents an outline of performance determinants in CrossFit^®^ that were investigated among 21 included studies. Based on this, we establish a guiding principle for training strategies and preparation of future competitions. In summary, the studies show high variety in the analyzed predictor variables and indicate broad discrepancies in the results. These findings underline the unknowable and diverse character of the exercise program, which resembles the demands of tactical populations. In the annual ‘CrossFit^®^ Games’, athletes have to perform their best way in an unforeseeable five-day physical challenge that includes numerous disciplines. Based on this, the ‘CrossFit^®^ Games’ claim to be the ultimate fitness challenge and elect the ‘The Fittest on Earth^®^’ [39]. This ideology raises the question whether we can at all predict competitive performance in this sport. From the CrossFit^®^ brands perspective, predictability is presumably not aspired, as they want the ‘CrossFit^®^ Games’ to be the ultimate unpredictable event to test physical fitness. Nevertheless, this systematic review aimed to identify and summarize predictors of performance across up-to-date literature, to enable sports scientists and coaches to develop specific training recommendations for the effective preparation of future competitions. To conclude, today’s literature still provides limited data to guide athletes in identifying the most important determinants for successful training and competition preparation. This review suggests focusing on body composition, aerobic capacity, and body strength first. Further, an appropriate pacing strategy and competition experience may be recommended for CrossFit^®^ performance prediction and enhancement. Future research is needed to verify these assumptions among both genders and should also address the effects of sports background and financial impact on performance. It is however still questionable, whether performance in unforeseeable events such as the ‘CrossFit^®^ Games’ is predictable at all.

## Figures and Tables

**Table 1:** Overview in predictors of performance outcomes for CrossFit^®^ athletes.

Reference	Data Collection	Sample (Gender)	Predictor	Predicted Performance	R-Squared (R^2^) or Correlation Coefficient ^a^ (r)
Mangine et al., 2021 [50]	Experimental data	11 CrossFit^®^ Open competitors (male = 5; female = 6)	Average round rate of a workout with multiple rounds (reps·s^−1^)	2016 CrossFit^®^ Open 16.2	R^2^ = 0.99
				2016 CrossFit^®^ Open 16.5	R^2^ = 0.94
				2016 CrossFit^®^ Open 16.1	R^2^ = 0.89
			Slowest round rate of a workout with multiple rounds (reps·s^−1^)	2016 CrossFit^®^ Open 16.3	R^2^ = 0.94
			Wall ball completion rate of a one round workout (reps·s^−1^)	2016 CrossFit^®^ Open 16.4	R^2^ = 0.89
Mangine et al., 2020 [51]	Experimental data	16 experienced (>2 years) athletes (male = 8; female = 8)	Body fat percentage (%)	2018 CrossFit^®^ Open 18.1	R^2^ = 0.89
				2018 CrossFit^®^ Open 18.3	R^2^ = 0.62
				2018 CrossFit^®^ Open 18.2a	R^2^ = 0.55
			Body density (kg·L^−1^)	2018 CrossFit^®^ Open 18.4	R^2^ = 0.77
				2018 CrossFit^®^ Open 18.5	R^2^ = 0.67
			Vastus lateralis cross-sectional area (cm^−1^)	2018 CrossFit^®^ Open 18.2b	R^2^ = 0.78
Meier et al., 2021 [52]	Reported data by questionnaire	162 CrossFit^®^ athletes (male = 66; female = 96)	Back squat (kg)	Clean and Jerk	R^2^ = 0.84
				Snatch	R^2^ = 0.76
Martínez-Gómez et al., 2020 [53]	Experimental data	15 male amateur CrossFit^®^ athletes	RSI (cm·ms^−1^), SJ (cm), and VO_2max_ (ml·kg^−1^·min^−1^)	Performance of the 2019 CrossFit^®^ Open ^b^	R^2^ = 0.81
Bellar et al., 2015 [54]	Experimental data	32 male CrossFit^®^ athletes	Age (years), CrossFit^®^ experience, WanT (watt), and VO_2max_ (ml·kg^−1^·min^−1^)	AMRAP workout (12 min)	R^2^ = 0.80
			CrossFit^®^ experience	FT workout (21-15-9)	R^2^ = 0.59
Butcher et al., 2015 [55]	Experimental data	14 experienced CrossFit^®^ athletes (male = 10; female = 4)	Total body strength (CrossFit^®^ Total in kg)	Grace	R^2^ = 0.77
				Fran	R^2^ = 0.42
Peña et al., 2021 [56]	Experimental data	10 experienced male CrossFit^®^ athletes	SJ (cm), CMJ (cm), RSI (cm·ms^−1^), snatch (kg), bench press (kg), and back squat (kg)	Simulated CrossFit^®^ competition with three benchmark workouts (Fran, Isabel, and Kelly)	R^2^ = 0.75
Feito et al., 2019 [57]	Experimental data	29 physical-active (advanced level trained) adults (male = 15; female = 14)	Repeated WanT performance	AMRAP workout (15 min)	R^2^ = 0.74
Dexheimer et al., 2019 [58]	Experimental data	17 experienced CrossFit^®^ athletes (male = 12; female = 5)	VO_2max_ (ml·kg^−1^·min^−1^)	Nancy	R^2^ = 0.68
			WanT (watt)	CrossFit^®^ Total	R^2^ = 0.57
			Back squat (kg)	Fran	R^2^ = 0.42
Dexheimer et al., 2020 [59]	Experimental data	17 trained males	Total body strength (CrossFit^®^ Total in kg)	Grace	R^2^ = 0.62
Martínez-Gómez et al., 2019 [60]	Experimental data	20 trained males	Back squat (% of body mass)	Performance of the CrossFit^®^ Open 2017 ^b^	R^2^ = 0.42
			Back squat (kg)		R^2^ = 0.38
Zeitz et al., 2020 [61]	Experimental data	22 trained participants (male = 13; female = 9)	VO_2max_ (ml·kg^−1^·min^−1^)	2019 CrossFit^®^ Open 19.1 (scaled)	R^2^ = 0.39
			Total body strength (CrossFit^®^ Total in kg)	Fran (modified)	R^2^ = 0.33
Tibana et al., 2021 [62]	Experimental data	17 experienced CrossFit^®^ athletes (male = 11; female = 6)	Tibana test (reps)	2020 CrossFit^®^ Open 20.5	r = −0.89 (r = −0.63) ^c^
				2020 CrossFit^®^ Open 20.2	r = 0.83 (r = 0.98) ^c^
				2020 CrossFit^®^ Open 20.3	r = 0.74 (r = 0.71 ^n.s.^) ^c^
				2020 CrossFit^®^ Open 20.1	r = −0.73 (r = −0.96) ^c^
				2020 CrossFit^®^ Open 20.4	r = 0.51 ^n.s.^ (r = 0.84) ^c^
Leitão et al., 2021 [63]	Experimental data	15 male CrossFit^®^ amateur athletes	Maximum reps of thrusters	Fran	r = −0.82
			2000 m row (s)		r = 0.67
			Thrusters (kg)		r = −0.61
			Maximum reps of pull-ups		r = −0.60
Barbieri et al., 2017 [64]	Use of public data	80 CrossFit^®^ Games 2016 finalist (male = 40; female = 40)	Fithy 50 (s)	Ranking in the CrossFit^®^ Games 2016	r = 0.77
			400 m sprint (s)		r = 0.69
			Snatch (kg)		r = −0.42
			Clean and Jerk (kg)		r = −0.39
Carreker et al., 2020 [37]	Experimental data	11 male experienced CrossFit^®^ athletes	Body fat percentage (%)	Murph	r = 0.72
Gómez-Landero et al., 2020 [65]	Experimental data	15 male CrossFit^®^ competitors	VO_2max_ (ml·kg^−1^·min^−1^)	Donkey Kong	r = −0.68
			Suprailiac skinfold		r = 0.71
			Sit-ups (reps)		r = −0.56
			Squat (kg)	Fran	r = −0.53
Cavedon et al., 2020 [66]	Experimental data	24 male CrossFit^®^ athletes	Appendicular LSTMI (kg/m^2^)	Fran	r = −0.65
			Amount of training (h/week)		r = −0.66
Schlegel et al., 2021 [67]	Reported data by questionnaire	Twenty best male Czechs in the CrossFit^®^ Open 2019 ranking	Snatch (kg)	Ranking in the CrossFit^®^ Open 2019	r = −0.61
			Clean and Jerk (kg)		r = −0.63
Mangine et al., 2022 [40]	Recorded data from publicly available online profile	220 randomly selected males from the top 1000 CFO 2020 athletes	Highest previous CrossFit^®^ Open rank	Overall and weekly ranking of the 2020 CrossFit^®^ Open	r = 0.26 to 0.39
			Individual regional appearances		r = −0.26 to −0.34
			Individual CrossFit^®^ Games appearances		r = −0.20 to −0.22
Klier et al., 2021 [68]	Reported data by online survey	149 CrossFit^®^ athletes (male = 68; female = 81)	Sleep quality by the Pittsburgh Sleep Quality Index (PSQI)	Hero-/Girl-Workouts	-
				Gymnastics	-

**Note.** ^a^ Pearson’s and Spearman’s correlation coefficient, respectively. ^b^ Overall performance in the ‘CrossFit^®^ Open’ (i.e., summing the final score of all WODs). ^c^ Values for women are given in brackets if the correlation coefficients are separated by gender. ^n.s.^, not significant.

## Data Availability

The datasets used and analyzed during the current study are available from the corresponding author on reasonable request.

## Supplementary Materials

The following supporting information can be downloaded at: https://www.mdpi.com/article/10.3390/sports11060112/s1, Table S1: Overview of Benchmark and CrossFit^®^ Open workouts [2,5,7].
