# Supplementary material for: CrossFit®: ‘Unknowable’ or Predictable?—A Systematic Review on Predictors of CrossFit® Performance
Source: Sports (Basel). 2023 May 30;11(6):112. doi: 10.3390/sports11060112 (PMC10304543; doi:10.3390/sports11060112)
Supplement: Supplementary file 1 [file sports-11-00112-s001.zip › sports-2402620-supplementary.pdf]

**Table S1.** Overview of Benchmark and CrossFit® Open workouts [2,5,7]. Certain data included in the following table are derived from CrossFit®. © Copyright CrossFit®, LLC 2022. All Rights Reserved.

| CrossFit®-Benchmark and CrossFit® Open workouts | | | | | | |
|---|---|---|---|---|---|---|
| **Girl-WODs** | **Cindy** | **Grace** | **Fran** | **Isabel** | **Kelly** | **Nancy** |
| | *20 min AMRAP of*<br><br>5 pull-ups<br>10 push-ups<br>15 air squats | *For time:*<br><br>30 clean and jerks (135/95 lb.) | *For time:*<br><br>*21-15-9 reps of*<br>thrusters (95/65 lb.)<br>pull-ups | *For time:*<br><br>30 snatches (135/95 lb) | *For time:*<br><br>*5 rounds of*<br>400-m run<br>30 box jumps (24/20 in)<br>30 wall ball shots (20/14 lb) | *For time:*<br><br>*5 rounds of*<br>400-m run<br>15 overhead squats (95/65 lb) |
| **Hero-WODs** | **Murph** | **Benchmark-WODs** | **CrossFit® Total** | **Donkey Kong** | **Fight Gone Bad** | **Filthy 50** |
| | *For time:*<br><br>1-mile run<br>100 pull-ups<br>200 push-ups<br>300 air squats<br>1-mile run | | *Total sum of the best from each lift:*<br><br>back squat<br>shoulder press<br>deadlift<br><br>*Three attempts for each lift* | *For time:*<br><br>*21-15-9 reps of*<br>Burpees<br>kettlebell swings (50/35 lb.)<br>box jumps (24/20-inch)<br><br>*Perform 6 Lunges after each exercise* | *Total reps:*<br><br>1-min wall balls (20/14 lb.)<br>1-min sumo deadlift high-pulls (75/55 lb.)<br>1-min box jumps (24/20-inch)<br>1-min push press (75/55 lb.)<br>1-min row<br>1-min rest<br><br>*Three rounds with a running clock* | *For time:*<br><br>50 box jumps (24/20-inch)<br>50 jumping pull-ups<br>50 kettlebell swings (35/26 lb.)<br>50 walking lunge steps<br>50 knees-to-elbows<br>50 push press (45/35 lb.)<br>50 back extensions<br>50 wall balls (20/14 lb.)<br>50 burpees<br>50 double-unders |
| **CrossFit® Open WODs** | | | | | | |
| **2016** | **16.1** | **16.2** | | **16.3** | **16.4** | **16.5** |
| | *20 min AMRAP of*<br><br>25-ft. overhead walking lunge (95/65 lb.)<br>8 burpees<br>25-ft. overhead walking lunge (95/65 lb.)<br>8 chest-to-bar pull-ups | *Beginning on a 4-min clock, complete AMRAP of*<br>25 toes-to-bars<br>50 double-unders<br>15 squat cleans (135/85 lb.)<br>*If completed before 4 min, add 4 min to the clock and proceed to*<br>25 toes-to-bars<br>50 double-unders<br>13 squat cleans (185/115 lb.)<br>*If completed before 8 min, add 4 min to the clock and proceed to* | | *7 min AMRAP of*<br><br>10 power snatches (75/55 lb.)<br>3 bar muscle-ups | *13 min AMRAP of*<br><br>55 deadlifts (225/155 lb.)<br>55 wall-ball shots (20/14-lb. ball to 10/9-ft. target)<br>55-calorie row<br>55 handstand push-ups | *For time:*<br><br>*21-18-15-12-9-6-3 reps of*<br>thrusters (95/65 lb.)<br>burpees |

| | | | | | | |
|---|---|---|---|---|---|---|
| | | 25 toes-to-bars<br>50 double-unders<br>11 squat cleans (225/145 lb.)<br>*If completed before 12 min, add 4 min to the clock and proceed to*<br>25 toes-to-bars<br>50 double-unders<br>9 squat cleans (275/175 lb.)<br>*If completed before 16 min, add 4 min to the clock and proceed to*<br>25 toes-to-bars<br>50 double-unders<br>7 squat cleans (315/205 lb.) | | | | |
| **2017** | **17.1** | **17.2** | | **17.3** | **17.4** | **17.5** |
| | *For time:*<br><br>*10 dumbbell snatches (50/35 lb.)*<br>*15 burpee box jump-overs (24/20-inch)*<br>*20 dumbbell snatches (50/35 lb.)*<br>*15 burpee box jump-overs (24/20-inch)*<br>*30 dumbbell snatches (50/35 lb.)*<br>*15 burpee box jump-overs (24/20-inch)*<br>*40 dumbbell snatches (50/35 lb.)*<br>*15 burpee box jump-overs (24/20-inch)*<br>*50 dumbbell snatches (50/35 lb.)*<br>*15 burpee box jump-overs (24/20-inch)* | *12 min AMRAP of alternating 2 rounds of*<br>50-ft. weighted walking lunge (50/35 lb.)<br>16 toes-to-bars<br>8 power cleans (50/35 lb.)<br>*Then, 2 rounds of*<br>50-ft. weighted walking lunge (50/35 lb.)<br>16 bar muscle-ups<br>8 power cleans (50/35 lb.) | | *Prior to 8:00, complete 3 rounds of*<br>6 chest-to-bar pull-ups<br>6 squat snatches (95/65 lb.)<br>*Then, 3 rounds of*<br>7 chest-to-bar pull-ups<br>5 squat snatches (135/95 lb.)<br>**Prior to 12:00, complete 3 rounds of*<br>8 chest-to-bar pull-ups<br>4 squat snatches (185/135 lb.)<br>**Prior to 16:00, complete 3 rounds of*<br>9 chest-to-bar pull-ups<br>3 squat snatches (225/155 lb.)<br>**Prior to 20:00, complete 3 rounds of*<br>10 chest-to-bar pull-ups<br>2 squat snatches (245/175 lb.)<br>*Prior to 24:00, complete 3 rounds of*<br>11 chest-to-bar pull-ups<br>1 squat snatch (265/185 lb.)<br>**If all repetitions are completed, time cap extends by 4 min.* | *13 min AMRAP of*<br>55 deadlifts (225/155 lb.)<br>55 wall-ball shots (20/14-lb. ball to 10/9-ft. target)<br>55-calorie row<br>55 handstand push-ups | *10 rounds for time of*<br>9 thrusters (95/65 lb.)<br>35 double-unders |
| **2018** | **18.1** | **18.2a** | **18.2b** | **18.3** | **18.4** | **18.5** |
| | *20 min AMRAP of* | *For time:* | *1-RM of* | *For time:* | *For time:* | *7 min AMRAP of* |

| | | | | | | |
|---|---|---|---|---|---|---|
| | 8 toes-to-bar<br>10 dumbbell hang clean and jerks (50/35 lb.)<br>14-calorie row | *1-2-3-4-5-6-7-8-9-10 reps of*<br>dumbbell squats (50/35 lb.)<br>bar-facing burpees | clean<br><br>*Time cap: 12 minutes to complete 18.2 and 18.2a* | *2 rounds of*<br>100 double-unders<br>20 overhead squats (115/80 lb.)<br>100 double-unders<br>12 ring muscle-ups<br>100 double-unders<br>20 dumbbell snatches (50/35 lb.)<br>100 double-unders<br>12 bar muscle-ups | 21 deadlifts (225/155 lb.)<br>21 handstand push-ups<br>15 deadlifts, (225/155 lb.)<br>15 handstand push-ups<br>9 deadlifts, (225/155 lb.)<br>9 handstand push-ups<br>21 deadlifts, (315/205 lb.)<br>50-ft. handstand walk<br>15 deadlifts, (315/205 lb.)<br>50-ft. handstand walk<br>9 deadlifts, (315/205 lb.)<br>50-ft. handstand walk | 3 thrusters (100/65 lb.)<br>3 chest-to-bar pull-ups<br>6 thrusters (100/65 lb.)<br>6 chest-to-bar pull-ups<br>9 thrusters (100/65 lb.)<br>9 chest-to-bar pull-ups<br>12 thrusters (100/65 lb.)<br>12 chest-to-bar pull-ups<br>15 thrusters (100/65 lb.)<br>15 chest-to-bar pull-ups<br>18 thrusters (100/65 lb.)<br>18 chest-to-bar pull-ups<br>*This is a timed workout. If you complete the round of 18, go on to 21. If you complete 21, go on to 24, etc.* |

| **2019** | **19.1** | **19.2** | **19.3** | **19.4** | **19.5** |
|---|---|---|---|---|---|
| | *15 min AMRAP of*<br>19 wall-ball shots (20/14-lb. ball to 10/9-ft. target)<br>19-cal. row | *Beginning on an 8-min clock, complete AMRAP of*<br>25 toes-to-bars<br>50 double-unders<br>15 squat cleans (135/85 lb.)<br>25 toes-to-bars<br>50 double-unders<br>13 squat cleans, (185/115 lb.)<br>*If completed before 8 min, add 4 min to the clock and proceed to:*<br>25 toes-to-bars<br>50 double-unders<br>11 squat cleans, (225/145 lb.)<br>*If completed before 12 min, add 4 min to the clock and proceed to:*<br>25 toes-to-bars<br>50 double-unders<br>9 squat cleans, (275/175 lb.)<br>*If completed before 16 min, add 4 min to the clock and proceed to:*<br>25 toes-to-bars<br>50 double-unders<br>7 squat cleans, (315/205 lb.) | *For time:*<br>200-ft. dumbbell overhead lunge (50/35 lb.)<br>50 dumbbell box step-ups (24/20-inch)<br>50 strict handstand push-ups<br>200-ft. handstand walk | *For time:*<br>*3 rounds of*<br>10 snatches (95/65 lb.)<br>12 bar-facing burpees<br>*Then, rest 3 minutes before continuing with 3 rounds of*<br>10 bar muscle-ups<br>12 bar-facing burpees | *For time:*<br>*33-27-21-15-9 reps of*<br>thrusters (95/65 lb.)<br>chest-to-bar pull-ups |
| **2020** | **20.1** | **20.2** | **20.3** | **20.4** | **20.5** |

| | | | | | |
|---|---|---|---|---|---|
| | *For time:*<br>*10 rounds of*<br>8 ground to overhead<br>(95/65 lb.)<br>10 bar-facing burpees | *20 min AMRAP of*<br>4 dumbbell thrusters (50/35 lb.)<br>6 toes-to-bar<br>24 double-unders | *For time:*<br>21 deadlifts (225 lb/155 lb)<br>21 handstand push-ups<br>15 deadlifts, (225 lb/155 lb)<br>15 handstand push-ups<br>9 deadlifts, (225 lb/155 lb)<br>9 handstand push-ups<br>21 deadlifts, (315 lb/205 lb)<br>50-ft. handstand walk<br>15 deadlifts, (315 lb/205 lb).<br>50-ft. handstand walk<br>9 deadlifts, (315 lb/205 lb).<br>50-ft. handstand walk | *For time:*<br>30 box jumps (24/20-inch)<br>15 clean and jerks (95/65 lb.)<br>30 box jumps (24/20-inch)<br>15 clean and jerks (135/85 lb.)<br>30 box jumps (24/20-inch)<br>10 clean and jerks (185/115 lb.)<br>30 single-leg squats<br>10 clean and jerks (225/145 lb.)<br>30 single-leg squats<br>5 clean and jerks (275/175 lb.)<br>30 single-leg squats<br>5 clean and jerks (315/205 lb.) | *For time:*<br>20 min to complete the following work in any order<br>40 muscle-ups<br>80-calorie row<br>120 wall ball shots (20/14-lb. ball to 10/9-ft. target) |

**Note.** Weights are given in brackets, separated by gender (male/female). As many rounds or repetitions as possible (AMRAP); Repetitions (Reps).
